# Mesenchymal Stem Cell Therapy in Nonhematopoietic Diseases

**DOI:** 10.1155/2015/676903

**Published:** 2015-03-01

**Authors:** Katherine Athayde Teixeira de Carvalho, Gustav Steinhoff, Juan Carlos Chachques

**Affiliations:** ^1^Stem Cell Research Laboratory, Pequeno Príncipe Faculty & The Pelé Pequeno Príncipe Research Institute, Silva Jardim 1632, 80.250-200 Curitiba, PR, Brazil; ^2^Department of Cardiac Surgery, Reference and Translation Center for Cardiac Stem Cell Therapy (RTC), University of Rostock, Schillingallee 35, 18.057 Rostock, Germany; ^3^Cardiac Bioassist, Pompidou Hospital, University of Paris Descartes, Laboratory of Biosurgical Research, Carpentier Foundation, University of Paris, Leblanc 56, 75.015 Paris, France

The extensive clinical experiments with stem cells, particularly in the treatment of oncohematological diseases, opened up the possibility of trying out stem cells with nonhematopoietic diseases. Mesenchymal stem cells (MSCs) subsequently emerged as a promising source for the regeneration and repair of various tissues in the treatment of a range of diseases due to their presence in all derived mesenchymal tissues in the adult solid organs as well as in mesoderm from embryonic tissue. This, plus their pluripotentiality and the fact that they were easily obtainable, meant that MSCs represented an important source for studies in regenerative medicine.

MSCs have been shown to be capable of differentiating in various types of mesoderm derived cells as well as ectoderm cells, such as skeletal muscle cells, neurons, cardiomyocytes, osteocytes, chondrocytes, and others. MSCs in specific cultivation conditions* in vitro* and in host transplanted tissue, being niche dependent, arouse clinical interest in stem-cell therapy for regeneration in nonhematopoietic tissue diseases and the prospect of creating a mesenchymal stem-cell bank for research and subsequent clinical use.

In the development of clinical models, always preceded by preclinical studies, there are various requirements to guarantee the safety of therapy with stem cells and their products, including the GMP procedures, cytogenetic control, identification and characterization of cells by immune cytometric analysis, and demonstration of their pluripotentiality for the prospective use of MSC for tissue regeneration in nonhematopoietic diseases [1]. In the study of MSCs, the intrinsic properties—molecular, immunocytochemistry, isolation, expansion, differentiation, and cryopreservation—have made great advances and need further discussion. The fact that MSCs do not express the antigens of histocompatibility means that they can be used in allogeneic transplantation. The development of methods for isolating the subpopulations of MSC fractions together with the perspective with this subpopulation could obtain better results than the total population in replacement therapy for correction of determined specific function [2].

Researchers and physicians are looking into the physiopathology of some diseases in light of the intrinsic cell conditions on the development of each disease and are proposing therapies based on the cells themselves or on cell-based products. Some diseases are triggered in their physiopathology by autoimmune mechanisms that could be stabilized with the paracrine effects of MSCs, such as the anti-inflammatory effect [3]; other diseases are triggered by the senescence of the cells, as in conditions of cellular apoptosis, and the MSC cells could act with the paracrine effects such as antiapoptosis and by cell replacement; there are some diseases which result from the loss of the production of certain molecules and MSCs could differentiate in cells capable of producing the molecule [4]; in other diseases, there is an interruption in signals between the tissue cells, and the MSC paracrine effects could promote the connections through the production of growth factors [5]. On the other hand, in ischemic diseases, MSCs could be differentiated in specific types of specialized cells, such as vessels and contractile cells, cardiomyocytes [6]; in genetic diseases, MSCs could ensure the delivery of genes for gene therapy [7] and could act in immunomodulation with vaccines [8].

This issue provides discussions of the points described above and takes a look into the future of cell therapy in nonhematopoetic diseases, showing that it is nearer than we expect! It is reality!

*Katherine Athayde Teixeira de Carvalho* *Katherine Athayde Teixeira de Carvalho* *Gustav Steinhoff* *Gustav Steinhoff* *Juan Carlos Chachques* *Juan Carlos Chachques*
